# Effectiveness and safety of ainuovirine plus lamivudine and tenofovir DF in virologically suppressed people living with HIV-1: the 48-week results of a multicenter, real-world study

**DOI:** 10.1128/aac.01108-25

**Published:** 2026-02-03

**Authors:** Shi Zou, Xiuhong Zhao, Qian Zhang, Yanling Xiao, Songjie Wu, Jie Liu, Yuting Tan, Qianhui Chen, Shihui Song, Miao Tan, Wei Guo, Chunmei Wang, Ke Liang

**Affiliations:** 1 Department of Infectious Diseases, Zhongnan Hospital of Wuhan University 89674, Wuhan, China; 2 Department of Dermatology, Shandong Public Health Clinical Center, Shandong University 12589, Shandong, China; 3 Qingshan District Center for Disease Control and Prevention 498598, Wuhan, China; 4 Department of Gastroenterology, Zhongnan Hospital of Wuhan University 89674, Wuhan, China; 5 Department of Nosocomial Infection Management, Zhongnan Hospital of Wuhan University 89674, Wuhan, China; 6 Department of Pathology, Zhongnan Hospital of Wuhan University 89674, Wuhan, China; 7 Department of Pathology, School of Basic Medical Sciences, Wuhan University 619505 , Wuhan, China; Chinese Academy of Medical Sciences & Peking Union Medical College, Beijing, China

**Keywords:** HIV, ainuovirine, efavirenz, virological suppression, lipid profile, weight gain

## Abstract

HIV-1 treatment has advanced with various antiretroviral regimens. Although efavirenz (EFV)-based regimens have been widely used, current guidelines recommend integrase strand transfer inhibitor (INSTI)-based therapy as first-line. However, INSTI may cause weight gain and adverse lipid changes, creating new unmet metabolic needs. Novel agents like ainuovirine (ANV), a non-nucleoside reverse transcriptase inhibitor (NNRTI), may offer effective virological suppression (VS) with improved tolerability, but their long-term real-world safety and effectiveness remain unclear. This was a multicenter, retrospective observational cohort study. Virologically suppressed adults on a tenofovir disoproxil fumarate (TDF)/3TC+EFV regimen were either switched to TDF/3TC+ANV (ANV group) based on the physician’s discretion or continued on TDF/3TC+EFV (EFV group). Baseline demographic and clinical data were collected, and participants were followed for 48 weeks. The primary effectiveness outcome was the proportion of patients achieving HIV-1 RNA levels below the limit of quantification (LOQ) at week 48. Secondary outcomes included absolute or percentage changes from baseline in CD4+ T-cell count, CD4+/CD8+ ratio. Key secondary safety outcomes included absolute changes from baseline in body weight, BMI, fasting lipid profiles (total cholesterol [TC], triglycerides [TG], high-density lipoprotein cholesterol [HDL-C], low-density lipoprotein cholesterol [LDL-C]), and parameters of liver and renal function. A total of 350 participants who completed the 48-week follow-up were included, comprising 170 patients in the ANV group and 180 patients who remained on EFV. At week 48, the proportion of VS was 96.5% in the ANV group and 96.1% in the EFV group (difference: 1.00 percentage points; 95% CI: –2.77 to 2.77), confirming non-inferiority. No significant differences in CD4+ T-cell recovery or CD4+/CD8+ ratios were observed. The ANV group experienced significantly less weight gain than the EFV group (estimated treatment difference [ETD]: –0.79 kg; *P* < 0.001). A corresponding trend in BMI change was observed but did not reach statistical significance. ANV also led to more favorable lipid changes, including a significant reduction in total cholesterol (ETD: –0.52 mmol/L; *P* < 0.001) and triglycerides (ETD: –0.83 mmol/L; *P* < 0.001) compared to EFV. Liver and renal function profiles remained stable in both groups. Switching from EFV- to an ANV-based regimen effectively maintained VS and led to improved metabolic parameters, including less weight gain and a more favorable lipid profile. As an alternative switch strategy, the ANV-based regimen may be a more beneficial option for people living with HIV (PLWH) who are at high risk of weight-related or dyslipidemia-associated comorbidities.

## INTRODUCTION

The introduction of antiretroviral therapy (ART) has dramatically transformed HIV-1 infection from a life-threatening disease into a manageable chronic condition. Although integrase strand transfer inhibitors (INSTIs) are recommended as the preferred first-line option in international guidelines due to their potent antiviral effectiveness and high genetic barrier to resistance (1, 2), non-nucleoside reverse transcriptase inhibitor (NNRTI)-based regimens, particularly efavirenz (EFV), are still widely used in many regions (3). This is especially true in low- and middle-income countries, where EFV-based regimens remain listed as alternative options in the latest WHO guideline (4). Additionally, accumulating evidence has raised concerns about significant weight gain, dyslipidemia, and other metabolic complications associated with second-generation INSTIs, particularly when combined with tenofovir alafenamide (5, 6). These emerging metabolic challenges highlight the need for alternative regimens that maintain virological effectiveness while offering improved safety and tolerability.

Ainuovirine (ANV), a newly developed NNRTI in China, has demonstrated promising outcomes across various stages of clinical development. Early-phase clinical studies established its potent antiviral activity and favorable preliminary safety profile (7). Subsequently, pivotal clinical trials in both treatment-naïve and virologically suppressed people living with HIV (PLWH) have demonstrated that ANV achieves a virological suppression (VS) rate comparable to EFV, while exhibiting a more favorable safety and tolerability profile with fewer adverse effects (AEs), particularly in terms of neuropsychiatric symptoms and lipid disturbances (8, 9). While these controlled trials offer crucial evidence of effectiveness and safety, post-marketing real-world evidence is essential for assessing the performance of ANV in diverse patient populations beyond the strict confines of clinical studies. Both single-center and multicenter setting studies have validated the effectiveness, safety, and unique benefits in lipid metabolism of the ANV-based regimen in the real world (10–13). This accumulating body of evidence suggests that ANV may offer a more favorable safety profile while maintaining effective VS.

Given the necessity of lifelong antiretroviral therapy and the limited evidence on switching to ANV in suppressed patients, we previously conducted a 24-week real-world study comparing ANV and EFV. We reported that switching to an ANV-based regimen was non-inferior to continuing an EFV-based regimen in maintaining VS and was associated with more favorable lipid profiles (10). Building upon these 24-week findings, the long-term durability of these benefits remains to be established. Therefore, this follow-up multicenter, real-world, retrospective cohort study was designed to evaluate and compare the 48-week effectiveness and safety of switching to an ANV-based regimen versus continuing an EFV-based regimen in virologically suppressed adults with PLWH.

## MATERIALS AND METHODS

### Study design

This study was designed as a multicenter, retrospective observational cohort analysis carried out across six designated HIV treatment centers in China (Table S1). The research protocol was approved by the Independent Ethics Committee of Wuhan Infectious Disease Hospital, and the ethics item number was KY-2023-15. All participants voluntarily provided written informed consent prior to enrollment in accordance with ethical requirements. Eligible subjects were HIV-1-infected adults aged 18 years or older, with documented evidence of VS. In addition, the study enrolled patients with HIV-1 RNA levels above the limit of quantification (LOQ) who were deemed eligible for switching to the ANV group, and those without evidence of treatment failure in the EFV group, as determined by the treating physician. The detailed inclusion and exclusion criteria for participant selection have been outlined in a previous study (see Table S2) (11).

Virologically suppressed adults living with HIV-1 were included in this study. Key inclusion criteria were (i) age ≥18 years; (ii) receiving a tenofovir disoproxil fumarate (TDF)/3TC+EFV regimen for at least 6 months prior to baseline; and (iii) having documented VS (HIV-1 RNA below the LOQ) for at least 6 months.

The exclusion criteria included participants with severe metabolic disorders, cardiovascular diseases, significant neurological or psychiatric conditions, and active opportunistic infections. In addition, women who were pregnant or breastfeeding were excluded, as well as those of childbearing age whose partners were either unable or unwilling to use effective methods of contraception. These exclusions reflect the cautious approach taken in clinical practice when introducing a new antiretroviral drug, for whom safety data in these specific populations are still being established.

### Participants

Participants who had previously received lamivudine (3TC), TDF, and EFV were required to have achieved VS, defined as an HIV-1 RNA level below the LOQ (see Table S3 for specific LOQ criteria).

#### ANV group

Patients in the ANV group were initially treated with a TDF/3TC+EFV regimen. They were subsequently switched to an ANV-based regimen, consisting of once-daily triple-drug therapy, either as ANV (75 mg × 2 tablets) + 3TC (300 mg × 1 tablet) + TDF (300 mg × 1 tablet), or a fixed-dose combination tablet of ANV 150 mg/3TC 300 mg/TDF 300 mg. Patients continued the ANV regimen for at least 48 weeks. The switch from EFV to ANV was made either due to intolerance to EFV-based therapy or at the discretion of the physician.

#### EFV group

Participants in the EFV group were identified from the National Free Antiretroviral Treatment Program Database in China. These individuals had been receiving an EFV-based regimen, consisting of once-daily triple-drug therapy with EFV (600 mg × 1 tablet) + 3TC (300 mg × 1 tablet) + TDF (300 mg × 1 tablet). They continued this EFV-based regimen for at least 48 weeks from baseline.

### Study procedures and follow-up

Participants were managed and followed according to the standard of care at each participating center. Following the baseline visit (week 0), clinical and laboratory assessments were scheduled per routine practice, typically at 12, 24, 36, and 48 weeks. At each follow-up visit, the following data were collected from electronic medical records:

- Clinical data: baseline demographic and comorbidities. Comorbidities were defined as physician-diagnosed chronic conditions, including diabetes mellitus, hypertension, cardiovascular disease, chronic kidney disease, or malignancy.
- Laboratory data: fasting lipid profile (total cholesterol [TC], triglycerides [TG], high-density lipoprotein cholesterol [HDL-C], low-density lipoprotein cholesterol [LDL-C]), liver and renal function tests (alanine aminotransferase, aspartate aminotransferase, creatinine), CD4+ and CD8+ T-cell counts, and HIV-1 RNA viral load.

HIV-1 RNA levels were measured using a real-time polymerase chain reaction assay at each center’s clinical laboratory. Fasting lipid profiles and T-cell counts were analyzed using standardized protocols across all sites (Cobas C702 Chemistry Analyzer, Roche Diagnostics) to ensure consistency. The 48-week time point was predefined as the primary endpoint for this analysis.

### Outcomes

The primary effectiveness outcome of the study was the proportion of patients achieving VS, defined as HIV-1 RNA levels below the LOQ at week 48. Secondary outcomes were consistent with those pre-specified in the study design and included (i) immunological changes: specifically, absolute and percentage changes in CD4+ T-cell count and CD4+/CD8+ ratio from baseline to week 48. (ii) Safety outcomes included absolute changes in body weight, BMI, fasting lipid profiles (TC, TG, HDL-C, LDL-C), and parameters of liver and renal function from baseline to week 48.

### Statistical analysis

All patients who met the inclusion criteria and completed baseline and follow-up assessments up to 48 weeks were included in the analysis. Continuous variables were presented as means with standard deviations (SD) or medians with interquartile ranges (IQR). Comparisons between groups were performed using the Student’s *t*-test or the Mann-Whitney U test, as appropriate. Categorical variables were expressed as frequencies (n, %) and compared using Fisher’s exact test or Pearson’s chi-square test, depending on data distribution. Estimates of difference and 95% CI for virological outcomes used the Cochran-Mantel-Haenszel weighted Miettinen and Nurminen method. As no stratification factors were prespecified in this retrospective study, the analysis was performed without strata. For the non-inferiority analysis, the two-sided 95% confidence interval for the difference in VS rates was calculated. Non-inferiority was concluded if the lower bound of the 95% CI was above the prespecified margin (–10 percentage points). To minimize potential confounding, baseline age, CD4+ T-cell count, and HIV-1 RNA status were included as covariates in the analysis of covariance (ANCOVA) models for efficacy and metabolic outcomes. All statistical tests were two-tailed, and a *P* value of <0.05 was considered statistically significant. All statistical analyses were conducted using IBM SPSS Statistics for Windows, version 27.0 (IBM Corp., Armonk, NY, USA).

## RESULTS

### Patient characteristics

**Fig 1:**
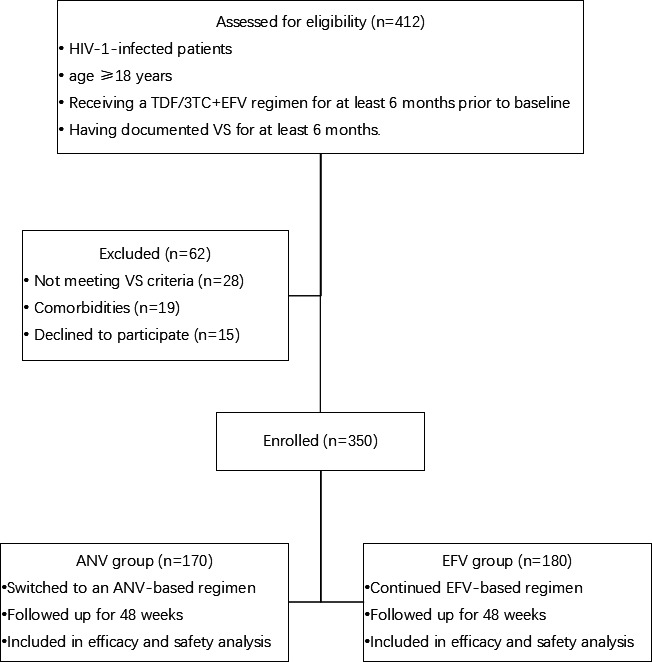
Participant disposition flow chart.

A total of 350 participants were enrolled, with 180 continued EFV and 170 switched from EFV to ANV. Figure 1 shows the flow of participant disposition. Detailed reasons for switching from EFV- to an ANV-based regimens were not consistently recorded across centers. The switch from EFV to ANV was made either due to intolerance to EFV-based therapy or at the discretion of the physician (11). Although participants in the ANV group were statistically younger than those in the EFV group, the difference was clinically minor, with fewer than 5% of patients aged ≥50 years in either group. The majority of the patients in each group (ANV group: 97.1%; EFV group: 96.7%) were male. The proportion of HIV RNA levels below the LOQ was 100% in the ANV group and 96.1% in the EFV group (*P* < 0.01). The mean CD4+ T-cell count was 611 cells/µL in the ANV group and 584 cells/µL in the EFV group. The proportions of participants with CD4 >500 cells/µL were 78.4% in the ANV group and 62.6% in the EFV group (*P* < 0.01). For details, see Table 1.

**TABLE 1:** Baseline demographic and clinical characteristics

Characteristic	Result for:		*P* value
	ANV group (*n* = 170)	EFV group (*n* = 180)	
Age, median years (range)	35 (31, 45)	33 (28, 39)	<0.01
Male, no. (%)	165 (97.1)	174 (96.7)	0.83
Weight (IQR), kg	74 (68, 79)	72 (65, 80)	0.13
BMI, mean (SD)	23.77 (2.6)	23.76 (2.5)	0.97
<18.5, no. (%)	6 (3.5)	3 (1.7)	0.13
18.5–24, no. (%)	79 (46.5)	108 (60.0)	
24–28, no. (%)	76 (44.7)	62 (34.4)	
≥28, no. (%)	9 (5.3)	7 (3.9)	
HIV-1 RNA, no. (%)			
Below the LOQ	170 (100.0)	173 (96.1)	<0.01
Above the LOQ	0 (0.0)	7 (3.9)	
CD4 count, mean (IQR), cells/μL	611 (448, 776)	584 (428, 754)	0.20
CD4 count strata, no.	162	171	
<200, no. (%)	3 (1.9)	9 (5.3)	0.10
200–500, no. (%)	32 (19.8)	55 (32.2)	<0.01
>500, no. (%)	127 (78.4)	107 (62.6)	<0.01
Comorbidities^*a*^, no. (%)	8 (4.7)	11 (6.1)	0.56

^*a*^ Diabetes, heart disease, hypertension, chronic kidney disease, and solid or hematological cancer.

### Effectiveness outcomes

**Fig 2:**
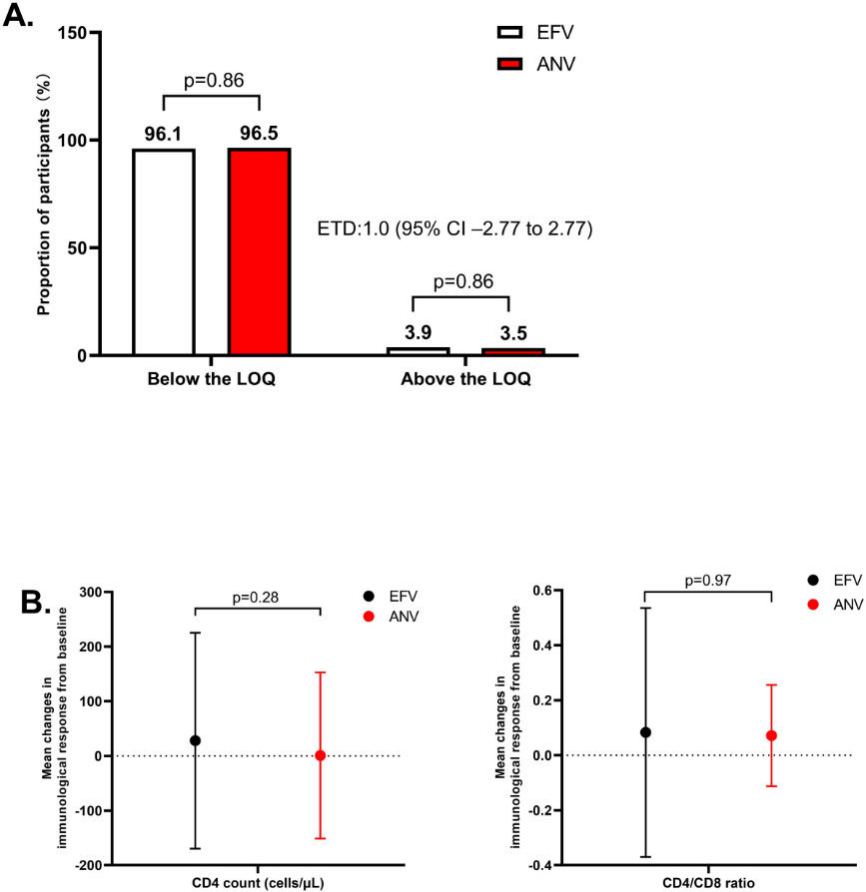
(**A**) Virological outcomes at 48 weeks. (**B**) Mean changes from baseline in CD4+ T-cell counts and CD4+/CD8+ ratios at week 48. Estimates for virological outcomes used the Cochran-Mantel-Haenszel weighted Miettinen and Nurminen method. ANV, ainuovirine; EFV, efavirenz. **P < 0.01, *P < 0.05.

More than 95% of participants maintained VS in both groups through 48 weeks. The results are depicted in Fig. 2A. By week 48, the proportions below the LOQ were 96.5% (164/170) in the ANV group and 96.1% (173/180) in the EFV group, with a difference of 1.00 percentage points (95% CI: –2.77 to 2.77), satisfying the predefined non-inferiority criterion. No participants met the criteria for genotypic resistance testing (confirmed HIV-1 RNA level exceeding 500 copies/mL) up to week 48. There were no significant differences in changes of CD4+ T-cell counts and CD4+/CD8+ ratios between baseline and week 48 between the ANV and EFV groups (Fig. 2B, *P* = 0.28 and 0.97). These findings indicate that the effectiveness of switching to an ANV-based regimen is comparable to continuing an EFV-based regimen by week 48.

### Changes from baseline in metabolism to week 48

**Fig 3:**
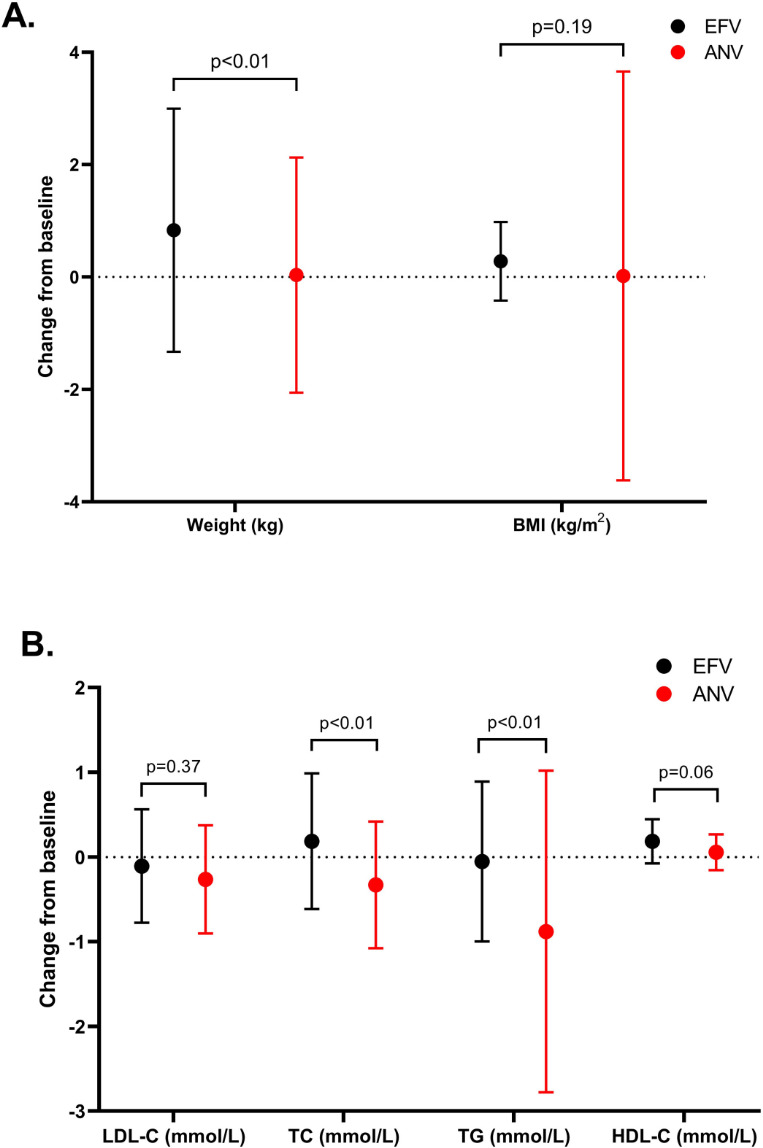
(**A**) Mean changes from baseline in body weight and BMI at week 48. (**B**) Mean changes from baseline in serum lipid parameters at week 4. Error bars indicate standard errors. ANV, ainuovirine; EFV, efavirenz; BMI, body mass index; TC, total cholesterol; TG, triglyceride; HDL-C, high-density lipoprotein; LDL-C, low-density lipoprotein. **P < 0.01, *P < 0.05.

Using analysis of covariance, Fig. 3 illustrates metabolic changes from baseline during a 48-week treatment period. The mean change in body weight was +0.04 kg in the ANV group, significantly less than the +0.83 kg increase in the EFV group (estimated treatment difference [ETD]: −0.79 kg; 95% CI: −1.12 to −0.46 kg; *P* < 0.01). Similarly, the change in BMI was +0.02 kg/m² in the ANV group versus +0.28 kg/m² in the EFV group (ETD: −0.26 kg/m²; 95% CI: −0.38 to −0.15 kg/m²; *P* = 0.19).

Regarding lipid profiles, by week 48, LDL-C decreased by –0.26 mmol/L in the ANV group and –0.11 mmol/L in the EFV group, with an ETD of –0.15 mmol/L (95% CI: –0.39 to 0.09; *P* = 0.37). TC decreased by –0.33 mmol/L in the ANV group but increased by 0.19 mmol/L in the EFV group, yielding an ETD of –0.52 mmol/L (95% CI: –0.71 to –0.33; *P* < 0.01). TG decreased more prominently in the ANV group (–0.88 mmol/L) than in the EFV group (–0.05 mmol/L), with an ETD of –0.83 mmol/L (95% CI: –1.21 to –0.45; *P* < 0.01). HDL-C increased in both groups, but the rise was greater in the EFV group (0.19 mmol/L) compared with the ANV group (0.06 mmol/L), with an ETD of –0.13 mmol/L (95% CI: –0.20 to –0.06; *P* < 0.01).

### Changes from baseline in biochemical indexes to week 48

**Fig 4:**
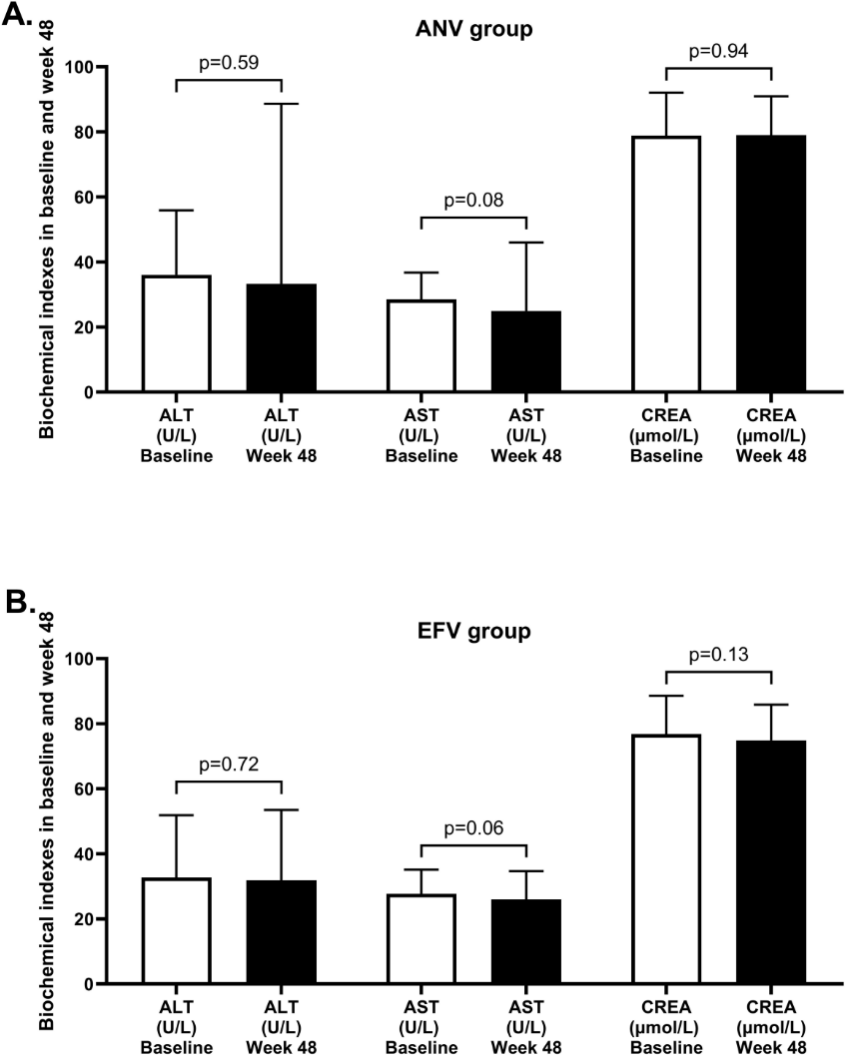
Changes from baseline in serum ALT, AST, and creatinine levels through 48 weeks. ALT, AST, and creatinine are shown for ANV (**A**) and EFV (**B**) groups at baseline and week 48. Data are presented as mean ± SE. ANV, ainuovirine; EFV, efavirenz; ALT, alanine aminotransferase; AST, aspartate aminotransferase; CREA, creatinine. **P < 0.01, *P < 0.05.

Figure 4 illustrates liver function and renal function changes from baseline to the 48-week treatment period. The results showed that both liver and renal function parameters remained stable during follow-up, and no clinically relevant differences were observed between groups. In addition, we analyzed the data and summarized the incidence of alanine aminotransferase (ALT)/aspartate aminotransferase (AST) elevations above 1.25×, 1.5×, and 2.5× ULN for both treatment groups. These results are provided in Table S4. The incidence of clinically significant liver enzyme elevations was low in both groups, with no statistically significant between-group differences.

## DISCUSSION

In our previous 24-week real-world cohort study (11), we demonstrated that switching from EFV to ANV maintained high levels of VS and improved metabolic outcomes compared with continuing EFV. In the current 48-week analysis, these benefits were sustained over a longer follow-up period, with VS rates of 96.5% in the ANV group and 96.1% in the EFV group, confirming non-inferiority. Importantly, high treatment adherence was observed in both groups, which may partly explain the consistently high VS rates and the absence of virological rebound or resistance. The durability of VS over 48 weeks underscores that the efficacy of ANV is not only comparable to EFV but also stable over time in the context of good adherence.

Our findings are consistent with evidence from other real-world studies and randomized controlled trials. For example, real-world analyses of treatment-naïve patients receiving ANV reported similar VS rates and favorable lipid changes compared with EFV at week 24 (10). Phase 3 randomized controlled trials further demonstrated that ANV was non-inferior to EFV at week 48 in treatment-naïve patients, with comparable VS and fewer adverse events (8). In addition, head-to-head comparisons between ANV and INSTI-based regimens indicated non-inferior virological efficacy (9). Together, these findings provide consistent evidence across different study designs that ANV can maintain long-term VS under real-world adherence conditions.

When comparing ANV with other ARVs, especially new-generation NNRTIs, our results highlight ANV’s competitive virological efficacy and metabolic profile. Rilpivirine (RPV)- and doravirine (DOR)-based regimens have been shown to achieve non-inferior VS compared with EFV in treatment-naïve populations and to maintain VS in switch studies, with improved tolerability (14–16). INSTIs, such as dolutegravir and bictegravir, also demonstrate potent antiviral activity and a high resistance barrier but are increasingly associated with weight gain and metabolic disturbances (5, 17, 18). In contrast, our study shows that ANV sustains VS while limiting weight gain and improving lipid profiles compared with EFV. This unique combination of durable antiviral efficacy, favorable adherence, and metabolic advantages positions ANV as a promising alternative among NNRTIs for long-term HIV management in virologically suppressed patients.

Immunologically, both treatment groups showed comparable improvements in CD4+ T-cell counts and CD4+/CD8+ ratios over 48 weeks. Although the ANV group showed a numerically higher median CD4+ T-cell counts at baseline, there was no statistically significant difference in immune recovery between the groups. This supports the notion that switching to ANV does not compromise immunologic benefit in the short to mid-term.

In recent years, metabolic considerations have gained increasing attention in ART management. Weight gain and associated cardiometabolic disturbances are emerging complications in PLWH receiving newer antiretroviral agents (19). Although the mean differences in body weight (approximately 0.8 kg) between groups may appear modest, such changes are clinically meaningful in the context of long-term cardiovascular health. Epidemiologic data in PLWH have demonstrated that even small, sustained reductions in weight gain are associated with lower risks of dyslipidemia, insulin resistance, and atherosclerotic cardiovascular disease (18). Therefore, ANV’s favorable body weight profile may contribute to cumulative cardioprotective effects over prolonged treatment duration. Interestingly, we observed a modest weight gain among patients who continued EFV through 48 weeks. This finding was somewhat unexpected, as EFV has traditionally been regarded as weight-neutral or even associated with lower weight gain compared with newer antiretroviral agents (5). Several factors may contribute to this observation. First, improvements in overall health status and immune recovery during long-term VS could lead to gradual increases in body weight independent of drug effect. Second, lifestyle and dietary changes, as well as the natural aging process of the cohort, may have contributed to this modest weight gain. Third, recent reports suggest that even non-INSTI regimens may not be entirely metabolically neutral over time, especially in real-world settings where patient heterogeneity is greater than in randomized trials (17, 18, 20). Additionally, our finding of minimal weight gain with ANV compared to EFV is consistent with its favorable metabolic profile. This profile is further highlighted by the recent SPRINT trial, in which an ANV-based regimen demonstrated significantly less weight gain compared to an elvitegravir-based INSTI regimen (9). While the comparator drugs differ, the collective evidence from both our study and the SPRINT trial robustly positions ANV as an antiretroviral with a low potential for weight gain, whether compared to a classic NNRTI or to certain INSTIs known to promote weight increase.

Lipid metabolism was another key differentiator in our cohort, with particular importance placed on LDL-C, a well-established surrogate endpoint for atherosclerotic cardiovascular disease risk. EFV has been historically associated with dyslipidemia, including elevations in TC, LDL-C, and TG, likely due to its mitochondrial toxicity and cytochrome P450 interactions (21, 22). Our results showed a trend toward a greater reduction in LDL-C in the ANV group compared to the EFV group (−0.26 mmol/L vs −0.11 mmol/L), although this difference did not reach statistical significance (*P* = 0.37). Crucially, the ANV group demonstrated a reduction in LDL-C, whereas the EFV group was associated with an increase in atherogenic lipids, such as TC. It is worth noting that while some NNRTIs, such as RPV, have been associated with minimal lipid changes, EFV consistently remains one of the NNRTIs with the most pronounced lipid elevations (14, 23). To better contextualize the lipid benefits of ANV within the modern NNRTI class, a comparison with DOR is insightful. Similar to our findings with ANV, DOR has demonstrated a lipid-neutral or even lipid-improving profile in clinical trials. For instance, the DRIVE-FORWARD and DRIVE-AHEAD studies showed that DOR-based regimens led to significantly more favorable lipid changes (reductions in TC and LDL-C) compared to DOR/RPV and EFV-based regimens, respectively (24). While head-to-head trials between ANV and DOR are lacking, the consistent trend of lipid reduction observed in our real-world cohort aligns with the lipid-neutral phenotype established for DOR. This suggests that ANV, like DOR, belongs to a newer generation of NNRTIs that provide potent virologic efficacy without the adverse lipid consequences historically associated with EFV. This shared characteristic makes both agents valuable options for patients at risk of cardiovascular disease, with the choice potentially being influenced by factors such as local availability, cost, and resistance profiles. Furthermore, future studies incorporating comprehensive lipidomics and metabolomic profiling are warranted to elucidate the precise metabolic mechanisms underlying ANV’s favorable lipid effects. Mass spectrometry-based quantification of specific lipid species, such as phospholipids, sphingolipids, and acylcarnitines, could provide a more detailed understanding of how ANV modulates fatty acid oxidation, cholesterol biosynthesis, and lipoprotein transport compared with EFV. These analyses will help determine whether the observed improvements in lipid parameters are driven by direct drug effects or secondary metabolic adaptations. Such mechanistic insights would be valuable for clarifying the metabolic pathways influenced by ANV and for optimizing long-term cardiovascular risk management in PLWH.

Safety was another important aspect of this comparison. Both regimens were generally well-tolerated, with no significant differences in liver or renal function markers over the 48-week period. It is worth noting that the risk of drug-induced liver injury is generally highest upon initial drug exposure in treatment-naïve individuals. Our cohort consisted of virologically suppressed patients who had already demonstrated tolerance to an EFV-based regimen, which may have selected for a population at lower risk for hepatic events. The observed stability in liver enzymes in both groups aligns with this context and supports the good hepatic safety profile of ANV in this stabilized population. This suggests that switching to ANV does not introduce new safety concerns, which is critical for patients with comorbid conditions. The lower incidence of neuropsychiatric and gastrointestinal AEs previously associated with EFV use, although not specifically quantified in our current study (11, 25).

This study has several limitations. First, its observational and retrospective design may introduce selection bias, and residual confounding cannot be fully excluded despite multi-center recruitment, uniform eligibility criteria, and adjustment for baseline age, CD4^+^ count, and HIV-1 RNA in ANCOVA models. Although the ANV group was slightly older with higher baseline CD4^+^ counts, these differences are unlikely to fully explain the more favorable lipid profile observed, though age-related changes in hepatic lipid metabolism may have contributed modestly. Future randomized or propensity-matched studies are needed to confirm ANV’s independent metabolic effects. Second, as a real-world study, no formal sample size calculation was performed; the cohort was sufficient for the primary non-inferiority endpoint but may be underpowered for some secondary outcomes. Third, while common laboratory and metabolic parameters were assessed, the study was not designed to detect rare adverse events, and patient-reported neuropsychiatric or gastrointestinal symptoms were not systematically captured, so the analysis focused on objective safety indices. Previous 24-week data suggested fewer such AEs with ANV, but prospective studies with standardized monitoring are needed for longer-term confirmation. Finally, the 48-week follow-up supports medium-term durability of VS and metabolic benefits, yet long-term sustainability remains unknown. Virologic failure was defined as >500 copies/mL, consistent with Chinese real-world practice; while less sensitive than the >200 copies/mL cutoff used in some trials, this threshold minimizes misclassification from transient low-level viremia and reflects programmatic standards.

### Conclusions

In this real-world cohort of virologically suppressed PLWH, switching to a TDF/3TC+ANV regimen maintained VS non-inferior to continuing a TDF/3TC+EFV regimen over 48 weeks. The switch to ANV was associated with cardiometabolic benefits, including significantly less weight gain and a more favorable lipid profile, notably reductions in TC and TG. Along with evidence from previous studies in both treatment-naïve and virologically suppressed individuals, the ANV-based regimen represents a promising treatment option and a viable alternative to EFV-based therapy. These attributes may contribute to improved long-term treatment adherence and cardiometabolic health in the management of HIV infection.

## Data Availability

The data sets used and/or analyzed during the current study are available from the corresponding author on reasonable request. All supporting research data have been deposited in the Zenodo repository and are publicly accessible via the permanent identifier 10.5281/zenodo.17812006.

## SUPPLEMENTAL MATERIAL

Supplemental material aac.01108-25-s0001.docx Tables S1 to S4.

ASM does not own the copyrights to Supplemental Material that may be linked to, or accessed through, an article. The authors have granted ASM a non-exclusive, world-wide license to publish the Supplemental Material files. Please contact the corresponding author directly for reuse.

## References

[1] DHHS. 2023. Guidelines for the use of antiretroviral agents in adults and adolescents living with HIV

[2] Ambrosioni J, Levi L, Alagaratnam J, Van Bremen K, Mastrangelo A, Waalewijn H, Molina J-M, Guaraldi G, Winston A, Boesecke C, Cinque P, Bamford A, Calmy A, Marzolini C, Martínez E, Oprea C, Welch S, Koval A, Mendao L, Rockstroh JK, EACS Governing Board. 2023. Major revision version 12.0 of the European AIDS clinical society guidelines 2023. HIV Med 24:1126–1136. doi:10.1111/hiv.13542 37849432

[3] Costa B, Vale N. 2022. Efavirenz: history, development and future. Biomolecules 13:88. doi:10.3390/biom13010088 36671473 PMC9855767

[4] Organization W H. 2021. Consolidated guidelines on HIV prevention, testing, treatment, service delivery and monitoring: Recommendations for a public health approach. Geneva 34370423

[5] Kumar S, Samaras K. 2018. The impact of weight gain during HIV treatment on risk of pre-diabetes, diabetes mellitus, cardiovascular disease, and mortality. Front Endocrinol (Lausanne) 9:705. doi:10.3389/fendo.2018.00705 30542325 PMC6277792

[6] Ruiz G, Suarez Robles M, Negredo E, Alcami J, Gonzalez-Ruano P, Duenas Gutierrez C, Moreno Guillen SMartinez-Sanz J. 2025. Metabolic complications after initiating BIC/FTC/TAF versus DTG + 3TC in ART-naive adults with human immunodeficiency virus (HIV): a multicenter prospective cohort study. Clin Infect Dis 81:263–270. doi:10.1093/cid/ciae645 40155359

[7] Peng Y, Zong Y, Wang D, Chen J, Chen Z-S, Peng F, Liu Z. 2023. Current drugs for HIV-1: from challenges to potential in HIV/AIDS. Front Pharmacol 14:1294966. doi:10.3389/fphar.2023.1294966 37954841 PMC10637376

[8] Su B, Gao G, Wang M, Lu Y, Li L, Chen C, Chen Y, Song C, Yu F, Li Y, et al.. 2023. Efficacy and safety of ainuovirine versus efavirenz combination therapies with lamivudine/tenofovir disoproxil fumarate for medication of treatment-naïve HIV-1-positive adults: week 48 results of a randomized controlled phase 3 clinical trial followed by an open-label setting until week 96. Lancet Reg Health West Pac 36:100769. doi:10.1016/j.lanwpc.2023.100769 37547039 PMC10398592

[9] Zhang F, Wu H, Cai W, Ma P, Zhao Q, Wei H, Lu H, Wang H, He S, Chen Z, Chen Y, Wang M, Wan W, Fu H, Qin H. 2024. Switch to fixed-dose ainuovirine, lamivudine, and tenofovir DF versus elvitegravir, cobicistat, emtricitabine, and tenofovir alafenamide in virologically suppressed people living with HIV-1: the 48-week results of the SPRINT trial, a multi-centre, randomised, double-blind, active-controlled, phase 3, non-inferiority trial. Lancet Reg Health West Pac 49:101143. doi:10.1016/j.lanwpc.2024.101143 39092318 PMC11293588

[10] Long H, He Q, Bi Y, Ke Y, Xie X, Zhao X, Tan S, Luo Y, Chen Z, Yu X, Li L. 2024. Efficacy and effect on lipid profiles of Ainuovirine-based regimen versus Efavirenz-based regimen in treatment-naïve people with HIV-1 at week 24: A real-world, retrospective, multi-center cohort study. Biosci Trends 18:176–186. doi:10.5582/bst.2024.01070 38684402

[11] Wang C, Yu X, Ke Y, Fu Y, Luo Y, Li Y, Bi Y, Chen X, Li L, Zhao X, Chen Z. 2024. Efficacy and effect on lipid profiles of switching to ainuovirine-based regimen versus continuing efavirenz-based regimen in people with HIV-1: 24-week results from a real-world, retrospective, multi-center cohort study. Antimicrob Agents Chemother 68:e0166823. doi:10.1128/aac.01668-23 38483175 PMC10989015

[12] Ma S, Xie X, Fu Y, Gan L, Yang X, Kong L, Li J, Long H. 2024. Clinical benefits of novel non-nucleoside reverse transcriptase inhibitors: a prospective cohort study. Immun Inflamm Dis 12:e1217. doi:10.1002/iid3.1217 38578026 PMC10996378

[13] Zhang Q, Chen Z, Wang Y, Peng Y, Tan S, Li Y, Cao G, Bignotti A, Wu S, Wang M. 2023. Impacts of ainuovirine-based and efavirenz-based antiretroviral therapies on the lipid profile of HIV/AIDS patients in southern China: a real-world study. Front Med 10:1277059. doi:10.3389/fmed.2023.1277059 PMC10800701 38259850

[14] Sharma M, Saravolatz LD. 2013. Rilpivirine: a new non-nucleoside reverse transcriptase inhibitor. J Antimicrob Chemother 68:250–256. doi:10.1093/jac/dks404 23099850

[15] Barchi V, Rindi LV, Iannazzo R, Massa B, De Simone G, Andreoni M, Sarmati L, Iannetta M. 2022. Doravirine/lamivudine/tenofovir disoproxil fumarate-induced hypertriglyceridemia in a newly diagnosed AIDS patient. AIDS 36:2231–2233. doi:10.1097/QAD.0000000000003370 36382442 PMC9698147

[16] Colombier MA, Molina JM. 2018. Doravirine: a review. Curr Opin HIV AIDS 13:308–314. doi:10.1097/COH.0000000000000471 29794817

[17] Norwood J, Turner M, Bofill C, Rebeiro P, Shepherd B, Bebawy S, Hulgan T, Raffanti S, Haas DW, Sterling TR, Koethe JR. 2017. Brief report: weight gain in persons with HIV switched from efavirenz-based to integrase strand transfer inhibitor-based regimens. J Acquir Immune Defic Syndr 76:527–531. doi:10.1097/QAI.0000000000001525 28825943 PMC5680113

[18] Wohl DA, Koethe JR, Sax PE, McComsey GA, Kuritzkes DR, Moyle G, Kaplan L, van Wyk J, Campo RE, Cohen C. 2024. Antiretrovirals and weight change: weighing the evidence. Clin Infect Dis 79:999–1005. doi:10.1093/cid/ciae191 38606799

[19] Sun L, He Y, Xu L, Zhao F, Zhou Y, Zhang L, Peng Q, Zhang H, Zhang Q, Cao T, Song Y, Wang S, Rao M, Jia X, Liu X, Zhou J, Ju B, Wang H, Liu J. 2022. Higher risk of dyslipidemia with coformulated elvitegravir, cobicistat, emtricitabine, and tenofovir alafenamide than efavirenz, lamivudine, and tenofovir disoproxil fumarate among antiretroviral-naive people living with HIV in China. J Acquir Immune Defic Syndr 91:S8–S15. doi:10.1097/QAI.0000000000003040 36094509

[20] Lagathu C, Béréziat V, Gorwood J, Fellahi S, Bastard J-P, Vigouroux C, Boccara F, Capeau J. 2019. Metabolic complications affecting adipose tissue, lipid and glucose metabolism associated with HIV antiretroviral treatment. Expert Opin Drug Saf 18:829–840. doi:10.1080/14740338.2019.1644317 31304808

[21] Tashima KT, Bausserman L, Alt EN, Aznar E, Flanigan TP. 2003. Lipid changes in patients initiating efavirenz- and indinavir-based antiretroviral regimens. HIV Clin Trials 4:29–36. doi:10.1310/f2v7-3r46-vx6j-241r 12577194

[22] Calmy A, Tovar Sanchez T, Kouanfack C, Mpoudi-Etame M, Leroy S, Perrineau S, Lantche Wandji M, Tetsa Tata D, Omgba Bassega P, Abong Bwenda T, et al.. 2020. Dolutegravir-based and low-dose efavirenz-based regimen for the initial treatment of HIV-1 infection (NAMSAL): week 96 results from a two-group, multicentre, randomised, open label, phase 3 non-inferiority trial in Cameroon. Lancet HIV 7:e677–e687. doi:10.1016/S2352-3018(20)30238-1 33010241

[23] Cohen CJ, Andrade-Villanueva J, Clotet B, Fourie J, Johnson MA, Ruxrungtham K, Wu H, Zorrilla C, Crauwels H, Rimsky LT, Vanveggel S, Boven K, THRIVE study group. 2011. Rilpivirine versus efavirenz with two background nucleoside or nucleotide reverse transcriptase inhibitors in treatment-naive adults infected with HIV-1 (THRIVE): a phase 3, randomised, non-inferiority trial. Lancet 378:229–237. doi:10.1016/S0140-6736(11)60983-5 21763935

[24] Moyle G, Meng F, Wan H, Sklar P, Plank RM, Lahoulou R. 2025. Brief report: resolution of neuropsychiatric adverse events after switching to a doravirine-based regimen in the open-label extensions of the DRIVE-AHEAD and DRIVE-FORWARD trials. J Acquir Immune Defic Syndr 99:81–86. doi:10.1097/QAI.0000000000003599 39748155 PMC11970612

[25] Treisman GJ, Soudry O. 2016. Neuropsychiatric effects of HIV antiviral medications. Drug Saf 39:945–957. doi:10.1007/s40264-016-0440-y 27534750

