# Supplementary material for: Effectiveness and safety of ainuovirine plus lamivudine and tenofovir DF in virologically suppressed people living with HIV-1: the 48-week results of a multicenter, real-world study
Source: Antimicrob Agents Chemother. 2026 Feb 3;70(3):e01108-25. doi: 10.1128/aac.01108-25 (PMC12959095; doi:10.1128/aac.01108-25)
Supplement: Supplemental material — Tables S1 to S4. [file aac.01108-25-s0001.docx]

**Table S1. The six centers participated in this study**

| No. | Centers | Location |
| --- | --- | --- |
| 1 | Shandong Public Health Clinical Center, Shandong University | Jinan |
| 2 | Wuhan Jinyintan Hospital, Tongji Medical College of Huazhong University of Science and Technology | Wuhan |
| 3 | Guangzhou Eighth People’s Hospital, Guangzhou Medical University | Guangzhou |
| 4 | GuiYang Public Health Clinical Center | Guiyang |
| 5 | The First Hospital of Changsha City, Xiangya School of Medicine of Central South University | Changsha |
| 6 | Yunnan Provincial Infectious Disease Hospital | Kunming |

**Table S2. Detailed inclusion and exclusion criteria**

| Criteria |  |
| --- | --- |
| Inclusion Criteria |  |
| 1 | Adult patients (age ≥ 18 years) with confirmed HIV-1 infection. |
| 2 | Had been receiving a stable antiretroviral regimen of Tenofovir Disoproxil Fumarate/Lamivudine + Efavirenz (TDF/3TC+EFV) for at least 6 months prior to baseline. |
| 3 | Achieved virological suppression (VS), defined as HIV-1 RNA level below the limit of quantification (LOQ) as per local assay, for at least 6 months. |
| 4 | Availability of complete medical records and key laboratory data (HIV-1 RNA, CD4+ count, lipid profile, liver and renal function tests) at baseline and during the 48-week follow-up period. |
| Exclusion Criteria |  |
| 1 | Presence of severe metabolic disorders (e.g., uncontrolled diabetes mellitus). |
| 2 | History of significant cardiovascular disease (e.g., coronary artery disease, heart failure). |
| 3 | Active, significant neurological or psychiatric conditions that could impair adherence or compliance. |
| 4 | Active opportunistic infections or other AIDS-defining illnesses at the time of enrollment. |
| 5 | Known history of resistance to any of the study drugs (TDF, 3TC, EFV, or ANV). |
| 6 | Pregnancy or breastfeeding. |
| 7 | Women of childbearing potential with partners who were unable or unwilling to use effective contraception throughout the study period. |
| 8 | Any other condition that, in the opinion of the investigator, would make the patient unsuitable for the study or non-compliant with the requirements. |

**Table S3. Definition of limit of quantification (LOQ) by six centers**

| No. | Institution | LOQ, copies/mL |
| --- | --- | --- |
| 1 | Department of Dermatology, Shandong Public Health Clinical Center | 20 |
| 2 | Department of Infection and Immunology with Chinese Integrative Medicine, Wuhan Jinyintan Hospital, Tongji Medical College of Huazhong University of Science and Technology | 100 |
| 3 | Infectious Disease Center, Guangzhou Eighth People's Hospital, Guangzhou Medical University | 20 |
| 4 | Department of Infectious Disease, GuiYang Public Health Clinical Center | 20 |
| 5 | Department of Infection and Immunology, The First Hospital of Changsha City, Xiangya School of Medicine of Central South University | 20 |
| 6 | Department of Outpatient, Yunnan Provincial Infectious Disease Hospital | 20 |

**Table S4: Incidence of ALT/AST elevations above ULN thresholds in baseline and week 48**

| Parameter | Group | >1.25× ULN, n (%) | >1.5× ULN, n (%) | >2.5× ULN, n (%) |
| --- | --- | --- | --- | --- |
| ALT | Baseline |  |  |  |
|  | ANV (n=170) | 8 (4.7%) | 3 (1.8%) | 0 (0.0%) |
|  | EFV (n=180) | 7 (3.9%) | 10 (5.6%) | 2 (1.1%) |
|  | Week 48 |  |  |  |
|  | ANV (n=170) | 3 (1.8%) | 8 (4.7%) | 1 (0.6%) |
|  | EFV (n=180) | 6 (3.3%) | 13 (7.2%) | 2 (1.1%) |
| AST | Baseline |  |  |  |
|  | ANV (n=170) | 7 (4.1%) | 1 (0.6%) | 0 (0.0%) |
|  | EFV (n=180) | 6 (3.3%) | 1 (0.6%) | 0 (0.0%) |
|  | Week 48 |  |  |  |
|  | ANV (n=170) | 3 (1.8%) | 0 (0.0%) | 1 (0.6%) |
|  | EFV (n=180) | 3 (1.7%) | 3 (1.7%) | 0 (0.0%) |
